# Parasitism of the Invasive Brown Marmorated Stink Bug, *Halyomorpha halys* (Hemiptera: Pentatomidae), by the Native Parasitoid, *Trichopoda pennipes* (Diptera: Tachinidae)

**DOI:** 10.3390/biology8030066

**Published:** 2019-09-14

**Authors:** Neelendra K. Joshi, Timothy W. Leslie, David J. Biddinger

**Affiliations:** 1 Department of Entomology and Plant Pathology, 217 Plant Science Building, University of Arkansas, Fayetteville, AR 72701, USA; 2 Department of Biology, Long Island University, 1 University Plaza, Brooklyn, NY 11201, USA; timothy.leslie@liu.edu; 3 Fruit Research and Extension Center, Entomology, Pennsylvania State University, 290 University Drive, Biglerville, PA 17307, USA

**Keywords:** invasive species, biological control, brown marmorated stink bug, parasitoid

## Abstract

The invasive brown marmorated stink bug, *Halyomorpha halys* (Hemiptera: Pentatomidae), has been an important agricultural pest in the Mid-Atlantic United States since its introduction in 1996. Biological control by native species may play an important role in suppressing *H. halys* populations and reduce reliance on chemical control. We collected *H. halys* adults in agricultural areas of five Pennsylvania counties over two years to examine the extent and characteristics of adult stink bug parasitism by *Trichopoda pennipes* (Diptera: Tachinidae), a native parasitoid of hemipterans. The overall parasitism rate (in terms of *T. pennipes* egg deposition) was 2.38 percent. Rates differed among counties and seasons, but not between years. Instances of supernumerary oviposition were evident, and eggs were more commonly found on the ventral side of the thorax, although no differences in egg deposition were found between males and female hosts. *T. pennipes* has begun to target *H. halys* adults in Pennsylvania and has the potential to play a role in regulating this pest. Adult parasitism of *H. halys* by *T. pennipes* should continue to be monitored, and landscape management and ecological pest management practices that conserve *T. pennipes* populations should be supported in agricultural areas where *H. halys* is found.

## 1. Introduction

The invasive brown marmorated stink bug, *Halyopmorpha halys* Stål (Hemiptera: Pentatomidae), was first found in Allentown, PA in the United States in 1996 [1] and has since spread to many states throughout the country, as well as to Canada, Europe and South America [2,3]. *H. halys*, which is native to Eastern Asia, has become an economically important pest of many crops in the U.S., especially tree fruit in the Mid-Atlantic states [3,4]. For example, in Eastern U.S. apples and peaches, *H. halys* was shown to damage up to 25% of fruit per tree [5], and losses in apple production exceeded $37 million in 2010 alone [3]. As a result, the frequency and intensity of insecticide applications to control *H. halys* in orchards has also increased substantially [6]. There are relatively few insecticides that provide reliable control of *H. halys* adults, which fly into the orchards from adjacent managed and unmanaged habitats in the late summer and fall close to harvest for apples and peaches. This further restricts insecticide control options to those with short pre-harvest label restrictions. The majority of insecticides have limited residual activity against the adults, whereas eggs and nymphs are easier to control [7]. As an agricultural pest, *H. halys* is a promising candidate for biological regulation by predators and parasitoids, since it spends the majority of the fruit-growing season in unmanaged, wooded habitats rather than in agricultural crops where pesticides can limit biological control [8].

In commercial agriculture settings, as well as other ecosystems, the decoupling of invasive pests from their native biocontrol agents is often thought to be the most probable mechanism by which invasive species become extremely successful pest species [9]. Classical biological control programs seek to correct this imbalance by importing co-evolved natural enemies from the countries of origin, but this technique has been called into question for impacts on non-target arthropods and further disruption of natural ecosystems [10,11,12,13]. It has been argued that invasion biologists and ecologists should consider responses of native biological control agents (parasitoids and predators) to invasive host species [14]. Native species of predators and parasitoids may play an important role in regulating populations of not only native host species, but also other related invasive and exotic host species. Such shifts in the preference of native natural enemies may influence the population dynamics and long-term establishment of exotic species [14]. Classical biological control may have the advantage of much more rapid control of the co-evolved exotic prey than native predators or parasitoids, which may take many years to adapt to new prey, but native biological control agents have the advantage of already being adapted to local environmental conditions and ecosystems.

While classical biological control of exotic pests in agriculture has met with some notable successes in the past, there have also been some failures where the introduced species negatively impacted non-target species [12]. It is these failures that have led to more stringent regulatory constraints in releasing exotic biological control agents [15]. Many exotic biological control introductions have been made with little knowledge or consideration of the biological control factors already present in the introduced ecosystem. Due to a tendency to focus on exotic biological control agents of exotic prey, there is often less information on the importance of native natural enemies in controlling populations of exotic species. However, in the area of tree fruit integrated pest management (IPM) in the U.S., there are several key examples in which the primary biological control agents of exotic pests – such as codling moth [*Cydia pomonella* (L.)], Oriental fruit moth [*Grapholita molesta* (Busck)], spotted wing drosophila [*Drosophila suzukii* (Matsumura)], and European red mite (*Panonychus ulmi* Koch)—are native predators or parasitoids that adapted to these exotic hosts over time [16,17,18]. 

This study was part of a large, multi-state group effort to survey for and characterize the impact of indigenous arthropod natural enemies that were attacking *H. halys* [19]. In the Mid-Atlantic region, there are several parasitoids of stink bugs that could potentially contribute to *H. halys* control. Here, we focus on field parasitism by *Trichopoda pennipes* Fab. (Diptera: Tachinidae) that was previously noted to parasitize this introduced pest in caged studies [20]. This common and widely distributed fly is found throughout North America and is reported to have three geographically isolated host strains that are important in the biological control of several species of Coreidae, Pentatomidae and Largidae [21]. While most parasitoids target the egg stage of stink bugs, *T. pennipes* targets the adults (and late-stage nymphs), a life stage that is more difficult to control chemically. In this study, we document and provided baseline data on adult *H. halys* parasitism rates (in terms of egg deposition on the host) in the field by *T. pennipes* in several counties of Pennsylvania. In addition, both the sex of the host and the location of the tachinid eggs on the host were recorded, since the location of where the eggs are laid is important to the successful penetration of the host cuticle by tachinids [22], and there has been speculation that the ventral thoracic pheromone glands of the male stink bugs are likely the main host finding mechanism [20]. 

## 2. Materials and Methods

### 2.1. Field Collection 

A total of 43 samples ranging from 21 to 868 *H. halys* adults were collected from orchards, vegetables, field crops and overwintering sites in buildings during the 2012 and 2013 growing seasons as part of an effort to establish laboratory colonies for pesticide bioassay work. Samples were taken from 11 locations spread across five Pennsylvanian counties: Adams, Allegheny (2013 only), Berks (2012 only), Franklin and Lancaster. Field-collected adults were maintained in screened cages where they were allowed to mate and lay eggs to start new colonies for bioassays. 

### 2.2. Oviposition Patterns

Spent adults were placed in plastic petri dishes and then examined for the presence of tachinid eggs and any resulting tachinid flies that emerged from these cadavers were collected and identified. For each parasitized individual, the sex of the host and the location of the tachinid eggs on the host were recorded. Records related to location of egg deposition indicated whether eggs were found on the dorsal or ventral surface, and whether eggs were on the thorax or the abdomen (in only rare instances was an egg found on the head of the host). Because multiple eggs on a single host may occur—despite only a single parasitoid ultimately surviving [23]—the number of eggs on each host was also recorded. 

*T. pennipes* eggs on *H. halys* hosts were identified according to the original description and drawings [24], photographs and descriptions [20], and from adults reared from parasitized hosts. Parasitized *H. halys* and reared *T. pennipes* adults are preserved and stored at the Penn State Frost Entomological Museum, University Park, PA or the Penn State Fruit Research and Extension Center, Biglerville, PA, U.S.

### 2.3. Data Analysis

Chi-square contingency tests were conducted to examine independence of parasitism frequency between years, among counties, and among seasons. Collection dates within years spanned three seasons—spring (Mar 20–Jun 20), summer (Jun 21–Sept 21), and fall (Sept 22–Dec 20)—and were grouped accordingly for seasonal analysis. The Holm’s sequential Bonferroni method was used for post hoc comparisons in parasitism rates among counties and seasons. One-sample *t*-tests were conducted to examine if parasitism rates (i.e., percent of tachinid eggs deposited on *H. halys*) were significantly different from the expected value of 50 percent for: (1) males and females; (2) dorsal and ventral surface; and (3) thorax and abdomen. Since *t*-tests were based on percentage values, samples with small numbers of total eggs found (i.e., ≤5) were excluded from the analysis. Lastly, a descriptive analysis of frequency of supernumerary oviposition was conducted.

## 3. Results

**Figure 1:**
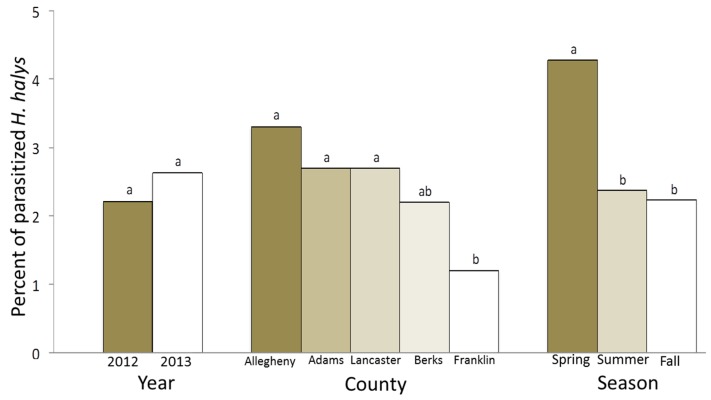
A comparison of *H. halys* adult parasitism rates between years, among counties, and among seasons in Pennsylvania (2012–2013).

During the two-year study, a total of 7857 *H. halys* adults were collected (2012: n = 4737; 2013: n = 3120) from 43 samples, of which 187 *H. halys* were parasitized (2.38%). Thirty of the 43 samples contained at least one parasitized individual. The percentage of parasitized individuals did not differ between the two years of the study [Figure 1; χ^2^ (1, N = 7587) = 1.37, *p* = 0.24]. Numerous *H. halys* adults were collected from multiple samples in Adams (n = 3332 individuals), Lancaster (n = 2178), and Franklin (n = 1636) counties, whereas fewer individuals were collected from a single sample taken in each of Allegheny (n = 121) and Berks (n = 590) counties. Parasitism rates differed among counties and ranged from 1.2 to 3.3 percent [Figure 1; (χ^2^ (4, N = 7587) = 14.07, *p* = 0.007]. *H. halys* adults were collected over three seasons: spring (n = 981), summer (n = 1477) and fall (n = 5399). Parasitism rates were higher in spring than in summer and fall [Figure 1; χ^2^ (2, N = 7587) = 19.33, *p* < 0.0001].

**Figure 2:**
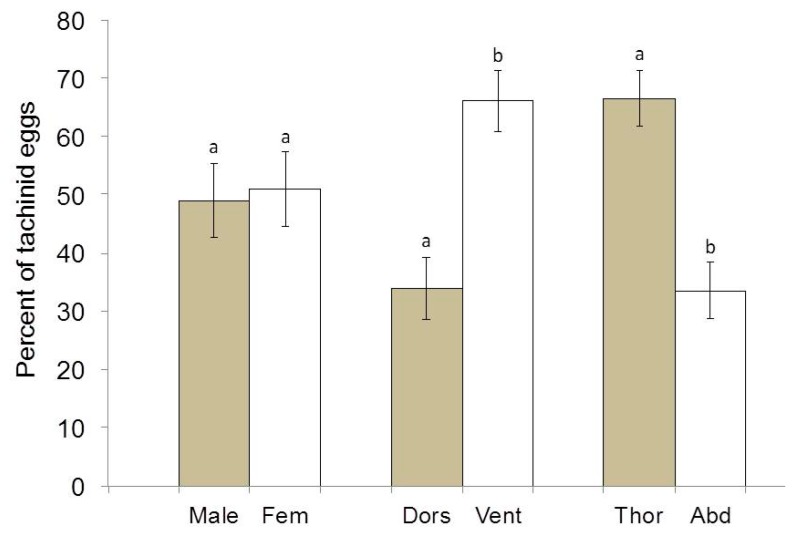
Mean percentage (+/− SEM) of *T. pennipes* eggs deposited on *H. halys* adults: (1) males versus females; (2) dorsum versus ventrum; and (3) thorax versus abdomen. ^a,b^ Different letters indicate significant differences in means.

A total of 259 eggs were found on the 187 parasitized *H. halys* adults. There was no difference in the percentage of eggs found on males versus females (Figure 2; t(15) = 0.15, *p* = 0.88). However, a greater percentage of eggs was found on the ventrum relative to dorsum (Figure 2; t(17) = 3.01, *p* = 0.008), and on the thorax relative to the abdomen (Figure 2; t(17) = 3.42, *p* = 0.003). 

**Figure 3:**
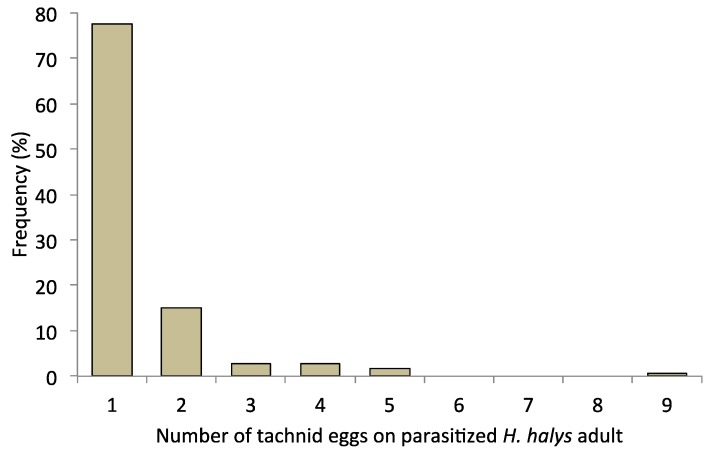
Frequency distribution of number of *T. pennipes* eggs found on parasitized *H. halys* adults.

Supernumerary oviposition (>1 egg/host) was observed on 22.4 percent of parasitized *H. halys* adults, whereas 77.8 percent had only one parasitoid egg (Figure 3). An average of 1.38 eggs was found on parasitized *H. halys* adults with up to nine eggs found on a single individual (Figure 3).

## 4. Discussion

Native predators and parasitoids have the potential to play an important role in the regulation of invasive species populations [14]. This study provides evidence that the native tachinid fly and known parasitoid of hemipterans, *T. pennipes*, is targeting up to 3.3% of adult brown marmorated stink bugs (*H. halys*) in Pennsylvanian agricultural landscapes. Other studies have recently provided evidence of other indigenous species exploiting *H. halys* [19] including egg parasitoids [25,26], egg predators [27], and nymph/adult predators [28,29,30]. Our findings, in combination with these other studies, reveal that native predators and parasitoids have begun to use the invasive *H. halys* as a food source or host, and could help regulate populations of this economically important pest. 

Although current adult parasitism rates are quite low, it is possible that the contribution of *T. pennipes* to the biological control of *H. halys* could increase over time. *T. pennipes* is multivoltine and a single adult female can lay hundreds of eggs. In addition, *T. pennipes* is already known to parasitize a range of other hemipteran pests [31]. Parasitism by *T. pennipes* can influence both lifetime fecundity and population growth of pests such as the southern green stink bug [*Nezara viridula* (L.)], although the effectiveness of *T. pennipes* can be limited due to the fact that the adult host is not immediately killed [32,33]. *T. pennipes* can also show host preference, and actually consists of a complex of biotypes or cryptic species across North America that specialize on regionally abundant pests, such as squash bugs [*Anasa tristis* (DeGeer)] [34] and bordered plant bugs [*Largus californicus* (Van Duzee)] in California [35]. It is thus worth monitoring if the exploitation of *H. halys* by *T. pennipes* increases in the Mid-Atlantic region of the U.S., and determining if this may represent the emergence of a new regional biotype that specializes on *H. halys*. 

*T. pennipes* populations should be conserved as part of the larger community of indigenous natural enemies in Mid-Atlantic agricultural landscapes to encourage biological control and potentially reduce reliance on insecticide use against *H. halys*. Native species of stink bugs in this area, which can cause catfacing (peaches), dimpling, scarring, discoloration and internal corking on tree fruits, are considered relatively minor pests, as their populations are held in check by a complex of parasitoids and generalist predators. Since *H. halys* exhibits similar characteristics and often moves into cropping areas from a surrounding unmanaged habitat, the best option for long-term population suppression may come from biological control in areas outside of the crop. However, in the absence of robust natural regulation, broad-spectrum insecticides, such as pyrethroids, are often needed for *H. halys* control. These products can impact the natural enemy community as a whole and can be quite disruptive to current IPM programs in tree fruit [6,36,37,38]. Similarly, pesticide drift to field margins or forest edges could negatively affect pollinators and natural enemies using these extra-orchard habitats. 

Our study found different parasitism rates among locations and this may have to do with *T. pennipes* abundance in the surrounding landscape. Certain plants, especially members of Asteraceae and Apiaceae, are known to support populations of beneficial flies such as syrphids and tachinids in agricultural areas [39]. For example, in Pennsylvania, *T. pennipes* emerges in late spring and has been observed using nectar sources from host plants such as wild carrot (*Dacaus carota* L.) and goldenrod (*Solidago* spp. L.) [39]. In general, agricultural landscapes with higher levels of plant diversity and/or habitat structure can provide shelter, favorable microclimatic conditions, and alternative food sources for natural enemies [40,41,42,43]. Therefore, providing nectar-rich resources in agricultural landscapes, whether through deliberate floral plantings or setting aside unmanaged areas in field margins, could potentially result in greater numbers of parasitoids and increased biological control (i.e., “parasitoid nectar provision hypothesis”) [43], and can be an important part of integrated pest and pollinator management (IPPM) programs in tree fruit orchards [44], where *H. halys* is an important pest and causes severe fruit infestation. 

In this study, we found significantly higher parasitism in *H. halys* adults during the spring season. Higher incidence of parasitism detected in the spring may have been an artifact of time, as eggs may have been deposited on spring-collected *H. halys* either during the previous year (prior to overwintering) or during the spring collection period. We also found significant patterns related to the location of egg deposition on *H. halys* adults. Studies suggest that *T. pennipes* locates stinkbug hosts by following the thoracic aggregation pheromones secreted by males [22,45] and field collections of southern green stinkbugs (*Nezara viridula*) found a greater number of parasitized males than females [46]. While our results did reveal a distinct preference for egg deposition on the ventral side of the thorax of *H. halys* adults, we did not find a preference for males. It is possible that *T. pennipes* used the male pheromones to locate *H. halys* aggregations and then laid eggs indiscriminately among males and females. Although not included in this analysis of adult parasitism, last instar nymphs of field-collected *H. halys* were also found with *T. pennipes* eggs, which supports previous findings that members of the Phasiine endoparasitoid group of Tachinidae are attracted to nymphal defensive allomones despite attacking mostly the adult stages [20,47].

## 5. Conclusions

Here, we provide evidence that *T. pennipes* has begun to target *H. halys* adults in agricultural landscapes of Pennsylvania, although at low rates. Parasitism rates varied across seasons and location. Although other studies suggest that *T. pennipes* more frequently targets male hosts, we found no difference in egg deposition on male and female hosts; however, *T. pennipes* oviposited more frequently on the ventral side of the thorax relative to other body regions of the host. *T. pennipes* is an effective and regionally specialized parasitoid of other hemipteran pests throughout the U.S. and thus has potential to contribute to the biological control of *H. halys*. In combination with targeted floral provisions and judicious use of insecticides, biological control by *T. pennipes* and other native parasitoids and predators, may play an increasingly prominent role in suppressing *H. halys* populations as part of IPM programs in the Mid-Atlantic region of the U.S.
